# PROCEDURES TO MEASURE MEAN AMBIENT DOSE EQUIVALENT RATES USING ELECTRET ION CHAMBERS

**DOI:** 10.1093/rpd/ncaa061

**Published:** 2020-06-03

**Authors:** F Leontaris, A Boziari, A Clouvas, M Kolovou, J Guilhot

**Affiliations:** 1 Department of Electrical and Computer Engineering, Aristotle University of Thessaloniki, GR-54124 Thessaloniki, Greece; 2 Greek Atomic Energy Commission, GR-15310 Agia Paraskevi, Greece

## Abstract

The capabilities of electret ion chambers (EICs) to measure mean ambient dose equivalent rates were investigated by performing both laboratory and field studies of their properties. First, EICs were ‘calibrated’ to measure ambient gamma dose equivalent in the Ionizing Calibration Laboratory of the Greek Atomic Energy Commission. The EICs were irradiated with different gamma photon energies and from different angles. Calibration factors were deduced (electret’s voltage drop due to irradiation in terms of ambient dose equivalent). In the field studies, EICs were installed at eight locations belonging to the Greek Early Warning System Network (which is based on Reuter-Stokes ionization chambers) for three periods, averaging 5 months each. In the same locations, *in situ* gamma spectrometry measurements were performed with portable germanium detectors. Gamma ambient dose equivalent rates were deduced by the *in situ* gamma spectrometry measurements and by soil sample analysis. The mean daily electret potential drop (in Volts) was compared with the mean daily ambient dose equivalent, measured with a portable HPGe detector and Reuter-Stokes high-pressure ionization chambers. From these measurements, ‘field’ calibration factors (electret’s voltage drop due to gamma radiation in terms of ambient dose equivalent) were deduced and found in very good agreement with the values deduced in Laboratory. The influence of cosmic radiation and the intrinsic voltage loss when performing long-term environmental gamma measurements with EICs, was estimated.

## INTRODUCTION

Electret ion chambers (EICs) are inexpensive, lightweight, commercially available, passive charge-integrating devices for accurate measurement of different radiations^(1–4)^. EIC are mainly used for short- or long-term radon measurements^(5,6)^. The device consists of a conducting plastic chamber containing an electret. Radon gas passively diffuses into the chamber through filtered inlets, and the alpha particles, emitted by the decay process of radon, ionize air molecules. Ions produced inside the chamber’s volume are collected onto the surface of the electret, causing a reduction of its surface charge**.** The electret voltage decreases proportional to the integrated radon concentration. A voltage reader is used to measure the electret’s surface voltage. Using appropriate calibration factors and the exposure time, the mean radon concentration can be calculated. However, with small modifications, EIC can be used for other type of radiations measurements. Particularly, EIC can become gamma monitors when sealed in a radon leak tight enclosure. In this case, the ionization of air molecules is due to the interaction of gamma radiation with the material of EIC and not due to the decay process of radon. Such modified devices have been used in comparison with TLDs in certain areas where low-energy gamma emitters, such as ^241^Am, were present. The TLDs were over-responding by ~ 50% to low-energy (60 keV) gamma associated with ^241^Am, whereas the EICs were responding accurately^(7)^. Radioisotope ^241^Am is one of the nine key radionuclides, which could be used in radiological dispersal devices (e.g. Dirty Bombs)^(8)^. Therefore, EIC could be useful for environmental monitoring following a radiological incident. Τhe EICs are also almost or total insensitive to normal environmental changes of temperature and pressure and consequently to seasonal variations^(4)^.

The use of EIC as gamma monitors is relative uncommon (in comparison to TLDs which are used in the majority of environmental gamma monitoring); consequently, the corresponding published work in this subject is limited^(4,7,9,10)^. The main scope of this work is to investigate the capabilities of EICs to measure ambient gamma dose equivalent ${H}^{\ast }(10)$ or ambient gamma dose equivalent rate dH^*^(10)/dt. The ambient equivalent dose ${H}^{\ast }(10)$ is a measurable quantity providing a conservative assessment of the effective dose, which quantifies the risk to human health associated to radiation exposure. This quantity was introduced by the International Commission on Radiation Units and Measurements (ICRU) back in 1985 (ICRU report 39^(11)^), and its use is also strongly recommended^(12)^ by ICRP, IAEA and other organizations and metrological institutes such as NIST, NPL, PTB, etc. In the European Union region, H^*^(10) must be used in area dosimetry according to EU Directive 96/29 EURATOM^(13)^. As the following, we use the symbol ${\dot{H}}^{\ast }$ as abbreviation for ambient dose equivalent rate, dH^*^(10)/dt, for simplicity.

## MATERIALS AND METHODS

EICs are supplied by Rad Elec Inc. The most commonly used EICs are available in six different configurations. Two different charged Teflon discs, named short-term electrets with high sensitivity and long-term (LT) electrets with low sensitivity, are available and can be associated with three different chambers named L (53 cm^3^), S (210 cm^3^) and H (960 cm^3^). In this work, the LST configuration (short-term electret associated with L chamber) was tested under laboratory and field conditions due to the following two reasons: the LST configuration is the only configuration for which information exists about leakage correction for long-term environmental gamma monitoring^(10)^ and it is the only one which was used for long-term environmental gamma monitoring^(10)^. The properties of EIC as gamma monitors were investigated by performing both laboratory and field studies.

### Laboratory studies

EICs were calibrated in terms of ambient dose equivalent ${H}^{\ast }(10)$ in the Ionizing Radiation Calibration Laboratory (IRCL) of the Greek Atomic Energy Commission (GAEC). IRCL is a secondary standard calibration laboratory. The EICs were irradiated at nine different photon energies (from 33 up to 1332 keV) at zero degree incidence (irradiation field perpendicular to the electret surface) and at 25 different angles (from 0° to 360°) for four different photon energies (mean X-ray energies 33 keV, 164 keV and gamma rays from ^137^Cs and ^60^Co radioactive sources). The ambient dose equivalent values were selected in order to obtain a voltage drop of about 40–60 V for each irradiation step, after which the voltage drop was measured. For each photon energy, five new, unused, EICs were used. In two of them, consecutive irradiations were performed (for each photon energy) in order to achieve a discharge from about 700 V (which was the initial voltage) down to 100–200 V. From the above measurements, calibration factors (electret’s voltage drop due to irradiation in terms of ambient dose equivalent) were deduced as a function of the incident’s photon energy and angle (energy and angular response).

### Field studies

**Figure 1:**
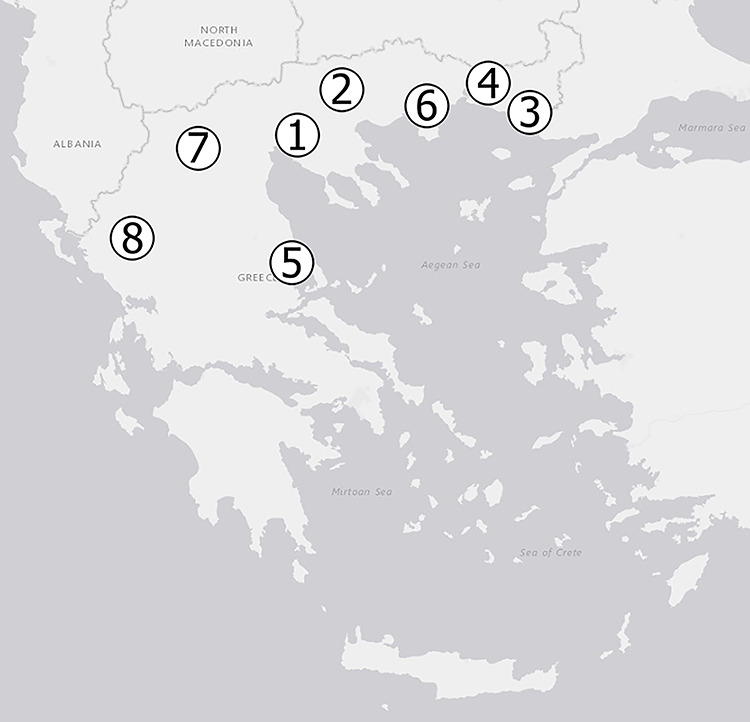
Location of the eight selected stations of the Greek early warning system network in which the field studies were performed.

Short-term electrets associated with L chambers were installed in different stations of the Greek Early Warning System Network which is administrated by the GAEC and consists of 24 Reuter-Stokes High-Pressure Ionization Chambers (HPIC) that can measure terrestrial and cosmic radiation. They are distributed all over Greece. In the present work, from a total of 24 locations, eight locations were selected. The eight selected stations are distributed mainly in Northern Greece (seven stations) and one of them in Central Greece as shown in Figure 1.

In each location, four–six EICs (LST configurations) were installed. The EICs by pair of two were sealed in a radon leak tight enclosure (mylar bags). The mylar bags were installed 1 m above soil near the Reuter-Stokes detector and remained for a period of about five months. The field measurements were repeated three times: November 2017–April 2018, April 2018–September 2018 and September 2018–March 2019.

The mean daily electret potential drop (in Volts) was compared with the mean daily ambient dose equivalent, measured with portable HPGe detector and Reuter-Stokes HPICs. From these measurements, ‘field’ calibration factors (electret’s voltage drop due to gamma radiation in terms of ambient dose equivalent) were deduced and compared with the Laboratory values obtained in the secondary standard calibration laboratory.

Table 1 shows the names of the locations, their code numbers as shown in Figure 1, their corresponding coordinates and altitude and the number of *in situ* gamma spectrometry measurements performed with the HPGe detector at each location.

**Table 1:** Names of the locations, their code numbers as shown in Figure 1, their corresponding coordinates and altitude and the number of *in situ* gamma spectrometry measurements with HPGe detector performed in each location.

No	Location	Coordinates	Alt. (m)	Number of *in situ* measurements
1	Thessaloniki	40° 37′ 54.12″ N	36	78
		22° 57′ 27.69″ E		
2	Serres	41° 04′ 36.42″ N	34	27
		23° 31′ 42.53″ E		
3	Alexandroupoli	40° 51′ 23.65″ N	4	6
		25° 56′ 51.81″ E		
4	Komotini	41° 08′ 02.55″ N	79	5
		25° 24′ 48.35″ E		
5	Volos	39° 22′ 42.77″ N	82	6
		22° 53′ 15.40″ E		
6	Kavala	40° 55′ 12.67″ N	4	4
		24° 37′ 12.79″ E		
7	Ptolemaida	40° 28′ 42.84″ N	651	4
		21° 43′ 34.16″ E		
8	Ioannina	39° 37′ 10.89″ N	487	4
		20° 50′ 48.70″ E		

### Dose rate measurements with Reuter-Stokes detectors

The dose rate probes are Reuter-Stokes spherical HPICs (Model RSS-131) using argon at ~25 MPa as counting gas. Each gamma dose rate detector has a sensitivity of less than 10 nGy h^−1^ for a 10-minute measurement. The energy range is 50 keV to 10 MeV; the measuring range is 10 nGy h^−1^ to 0.1 Gy h^−1^ with an accuracy of ±5% for the range between 10 nGy h^−1^ to 0.01Gy h^−1^ and ±7% above 0.01 Gy h^−1^. The directional response is ±2% over an angle of 4π. Each detector is coupled to a tipping bucket rainwater gauge model Young, Traverse, MI, USA. The latter device is also connected to the local data logger and modem, allowing registration and online consultation of pluviometric data. Gamma dose rate is calculated on 10-minute intervals, and data are stored in 1-hour intervals during normal periods and 10-minute intervals during emergencies (‘intensive mode’). Online computers placed at GAEC allow the evaluation of signal from the systems before eventually alerting the emergency planning offices. The Reuter-Stokes ionization chambers (of the Greek Early Warning System Network) measure in terms of exposure rate (μR h^−1^). In a recent work^(14)^, the detectors were recalibrated to measure in terms of ambient dose equivalent rate. With this recalibration, two major concerns were corrected^(14)^: (1) any calibration issue of the Reuter Stokes HPIC detectors due to their home-made calibration in terms of exposure and (2) the well-known over response^(15)^ of the Reuter-Stokes HPIC detectors to cosmic radiation.

### Gamma radiation measurements with portable HPGe detectors

Portable HPGe detectors with a 35 and 40% relative efficiency were used for the *in situ* gamma spectrometry measurements. The duration of each measurement was 2000 s. The methodology used for the derivation of the gamma dose rates from the *in situ* gamma ray spectra is the one introduced by Beck *et al.*^(16)^ and is called the ‘Peak Area Method’. Based on this method, Helfer and Miller^(17)^ derived simple calibration factors (for the outdoor measurements) which convert the measured full absorption peak count rate to activity in the soil and dose rate in air. The ambient dose equivalent rate ${\dot{H}}^{\ast }$ due to terrestrial component is easily deduced from the *in situ* gamma spectrometry measurements using the specific activity to ${\dot{H}}^{\ast }$ conversion coefficients calculated by Lemercier *et al.*^(18)^. In addition, slices (layers) of soil with a horizontal area of 38 cm^2^ and a height of 5 cm were collected in each location down to a 20-cm depth. The activity of each soil layer due to natural gamma emitters and ^137^Cs (from Chernobyl accident) was measured by standard gamma spectroscopy with a 50% relative efficiency HPGe detector. From the measured activities, gamma dose rates 1 m above the soil surface were indirectly deduced using the conversion coefficients calculated by Lemercier *et al.*^(18)^ and compared with the gamma dose rates deduced by the *in situ* gamma spectrometry measurements in order to validate the accuracy of these measurements. The calibration factors reported previously^(18)^ are valid if the soil is homogeneously distributed around the detector in a large ring. Natural gamma emitters are, in principle, homogeneously distributed around the detector. Most (85%) of the gamma radiation 1 m above soil comes within a soil radius of 10 meters^(17)^. Soil moisture may also alter measured dose rate values (about 1% increase of water content in soil corresponds to 1% decrease of the gamma dose rate compared to dry soil).

## RESULTS AND DISCUSSION

### Laboratory calibration results

**Figure 2:**
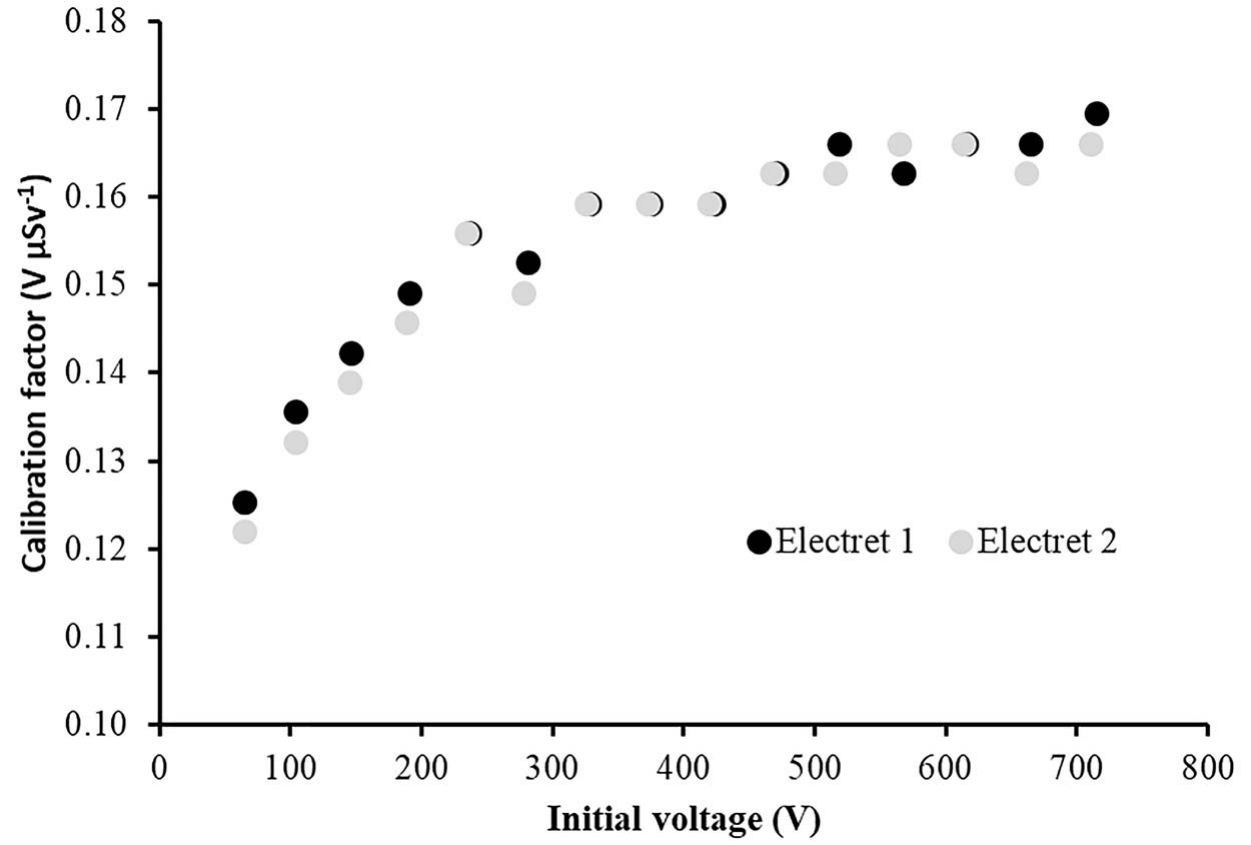
Calibration factors (electret potential lowering per μSv) for LST electret ionization chambers irradiated with Radiation Quality N-40 (33 keV) X-rays at zero-degree incidence with an ambient dose equivalent value for each irradiation of 295 μSv.

**Figure 3:**
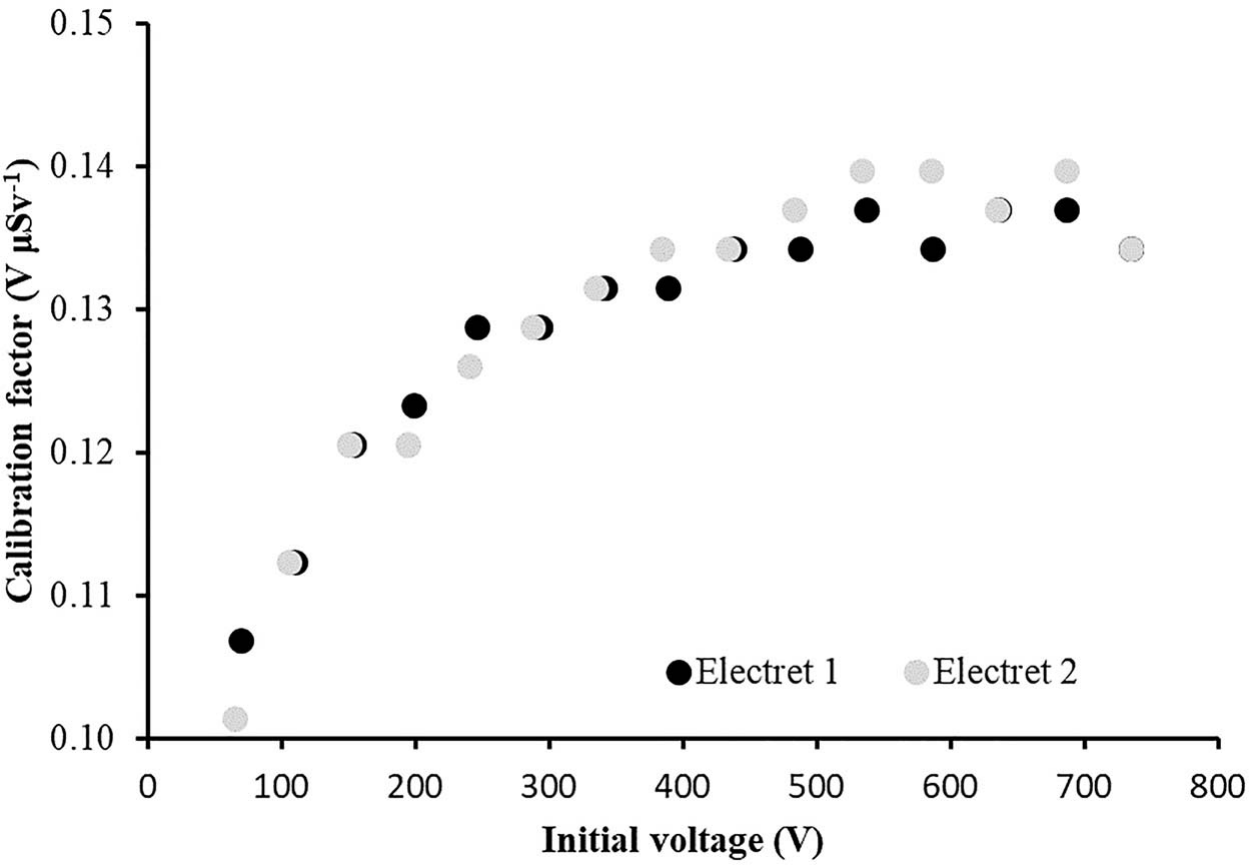
Calibration factors (electret potential drop per μSv) for LST electret ionization chambers irradiated with Radiation Quality N-200 (164 keV) X-rays at zero-degree incidence with an ambient dose equivalent value for each irradiation of 365 μSv.

**Figure 4:**
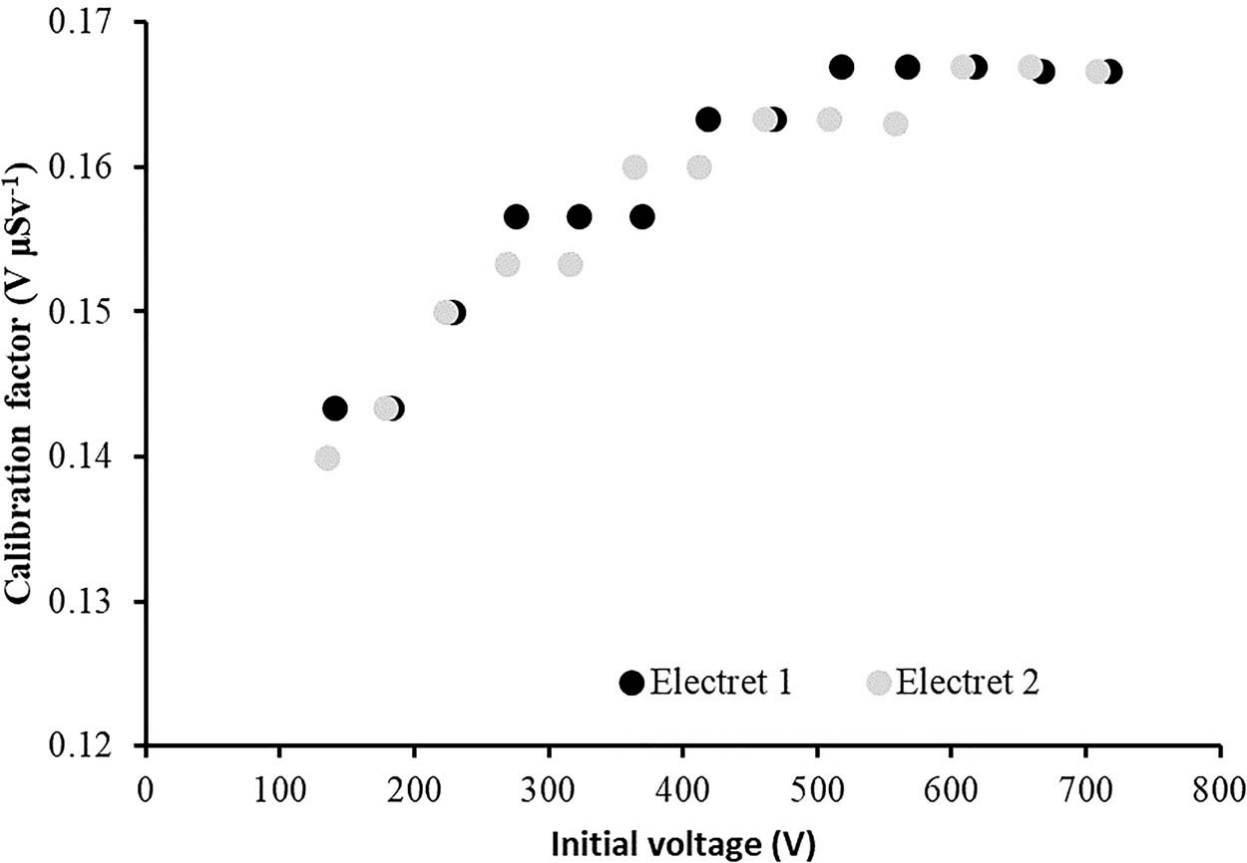
Calibration factors (electret potential lowering per μSv) for LST electret ionization chambers irradiated with gamma rays (point source 137Cs) at zero-degree incidence with an ambient dose equivalent value for each irradiation of 300 μSv.

**Figure 5:**
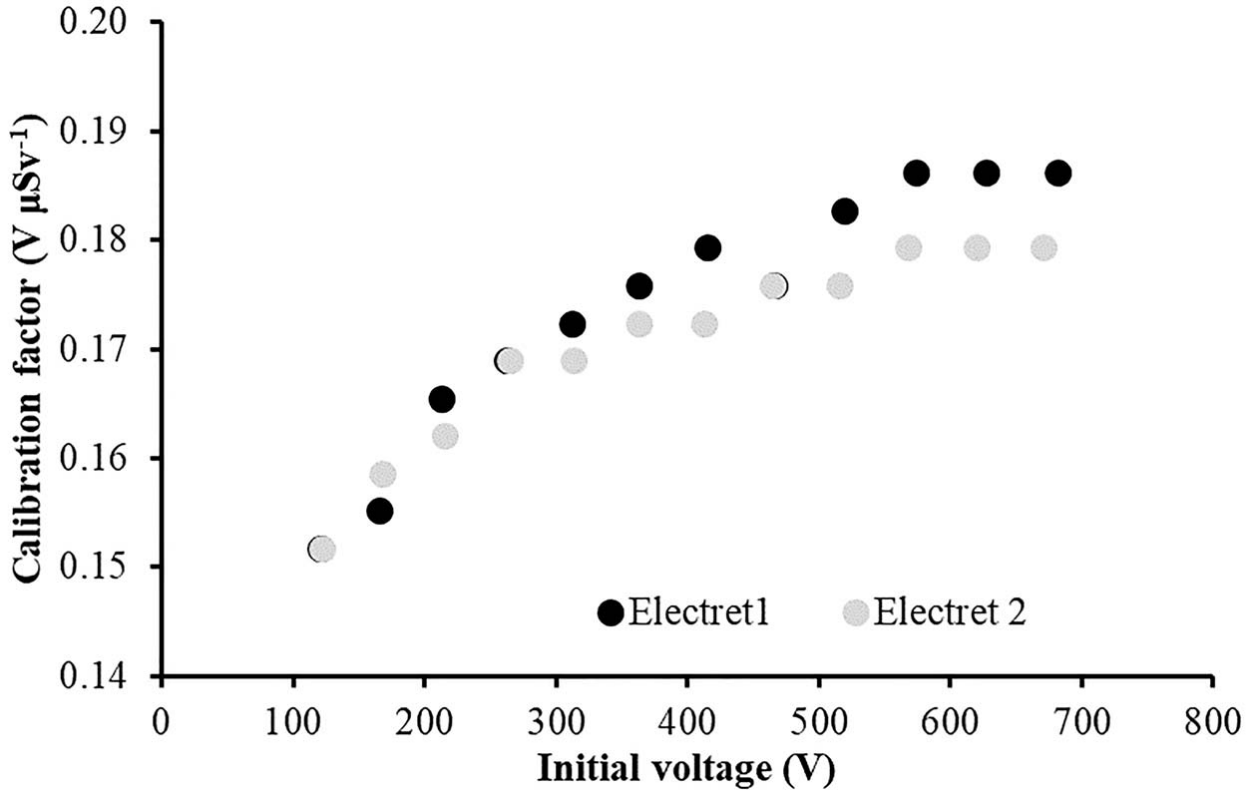
Calibration factors (electret potential lowering per μSv) for LST electret ionization chambers irradiated with gamma rays (point source ^60^Co) at zero-degree incidence with an ambient dose equivalent value for each irradiation of 290 μSv.

The calibration factors (electret’s voltage drops due to irradiation in terms of ambient dose equivalent) in V μSv^−1^ as a function of the initial voltage of the electret for four different irradiation energies are shown in Figures 2–5. The irradiation field was perpendicular to the electret surface (zero-degree incidence). It is clearly observed, in Figures 2–5, that no practical difference between the calibration factors measured by the two electrets exists. In addition, the calibration factors for a given irradiation energy depend to the initial electret voltage. However, for initial electret voltage higher than 450 V, the calibration factors seem to be independent of the initial electret voltage. New (unused) electrets have initial voltage more than 700 V. In case new, electrets are used for environmental gamma monitoring purposes; it is advisable to use them up to electret potential lowering of 250 V, so the final electret voltage (after irradiation) is 450 V.

In Table 2, the calibration factors (electret’s voltage drop due to irradiation in terms of ambient dose equivalent) in V μSv^−1^ for different irradiation energies (at zero-degree incidence) are shown.

**Table 2:** Calibration factors (electret’s voltage drop due to irradiation in terms of ambient dose equivalent) in V μSv^−1^ for different irradiation energies (at zero-degree incidence).

Radiation quality	Energy (keV)	Number of measurements	Calibration factors (V μSv^−1^)
N-40	33	17	0.164 ± 0.017
N-60	48	5	0.152 ± 0.016
N-80	65	5	0.148 ± 0.015
N-100	83	5	0.139 ± 0.014
N-120	100	5	0.138 ± 0.014
N-150	118	5	0.142 ± 0.015
N-200	164	8	0.141 ± 0.015
Cs-137	661.6	10	0.166 ± 0.017
Co-60	1173, 1332	13	0.181 ± 0.019

**Figure 6:**
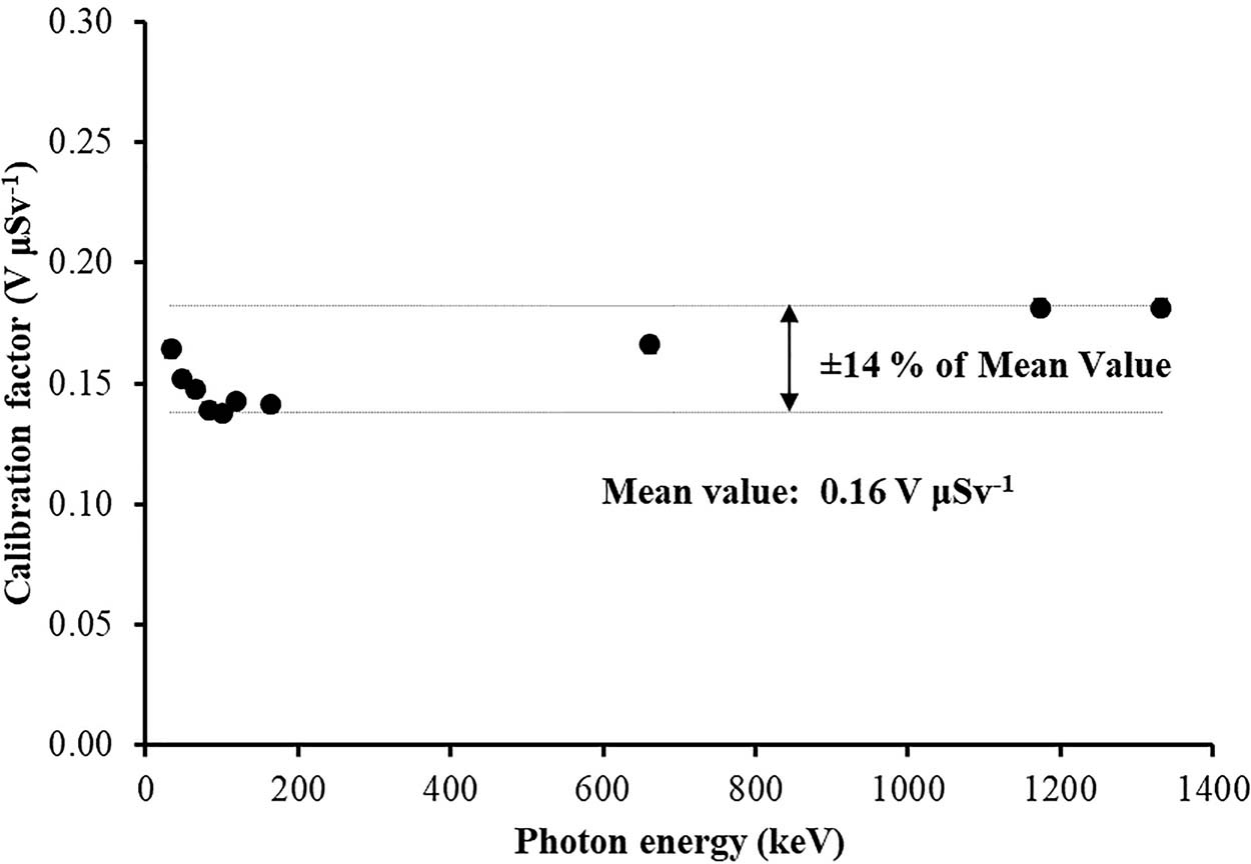
Irradiation photon energy dependence (at zero-degree incidence) of the calibration factors (electret’s voltage drop due to irradiation in terms of ambient dose equivalent) in V μSv^−1^.

In Table 2 and Figure 6, a small energy dependence of the calibration factors is observed with a mean value of CF = 0.16 V μSv^−1^. All CF values are within a range of 14% of the mean CF value (Figure 6).

**Figure 7:**
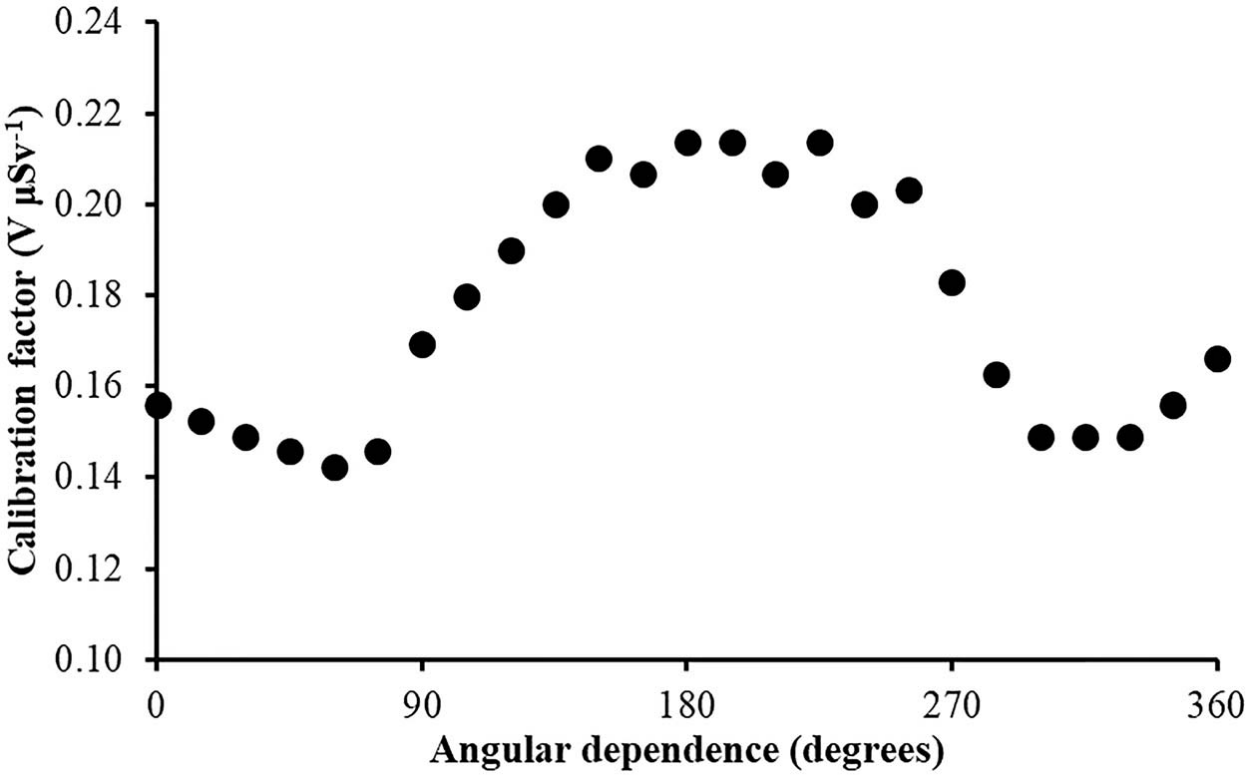
Angular dependence of calibration factors (electret potential drop per μSv) for LST electret ionization chambers irradiated with Radiation Quality N-40 (33 keV) X-rays (with an ambient dose equivalent value for each irradiation of 295 μSv).

**Figure 8:**
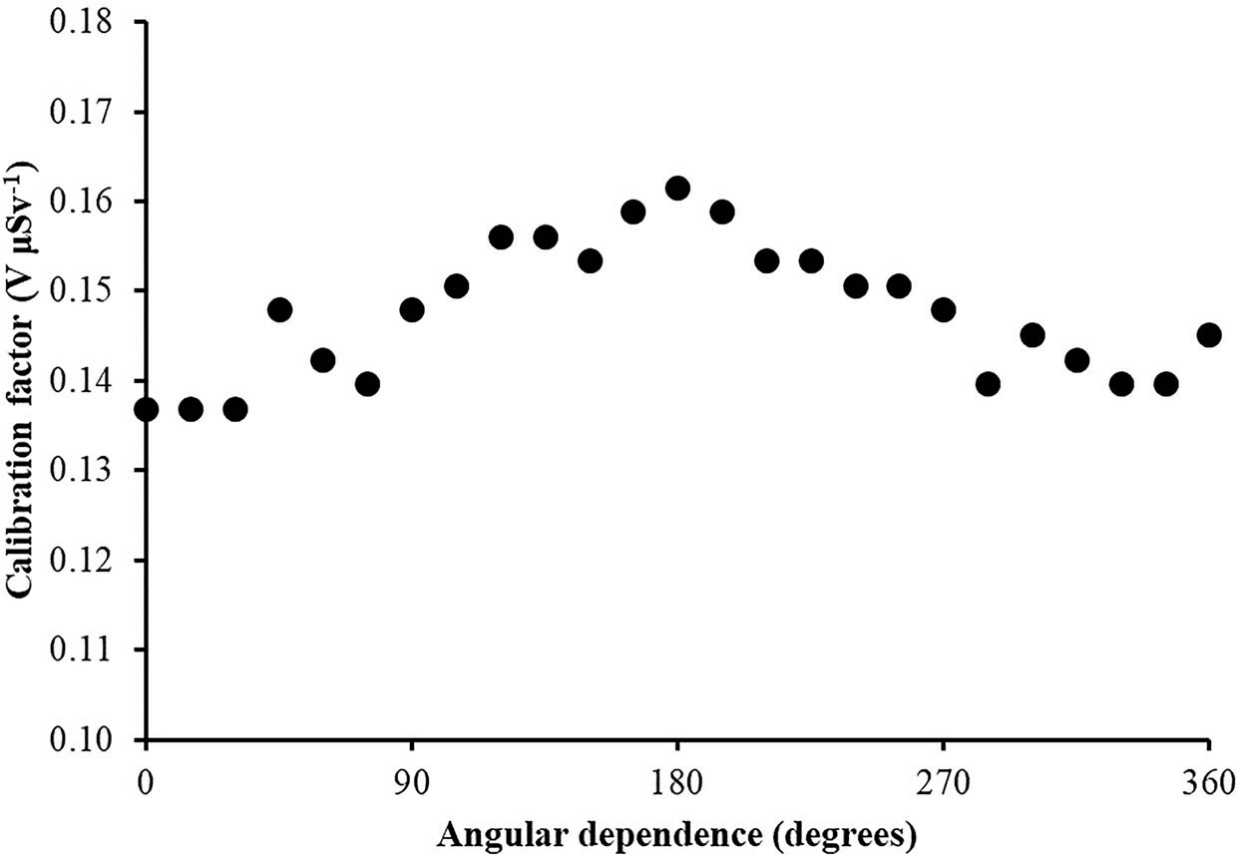
Angular dependence of calibration factors (electret potential drop per μSv) for LST electret ionization chambers irradiated with Radiation Quality N-200 (164 keV) X-rays (with an ambient dose equivalent value for each irradiation of 365 μSv).

**Figure 9:**
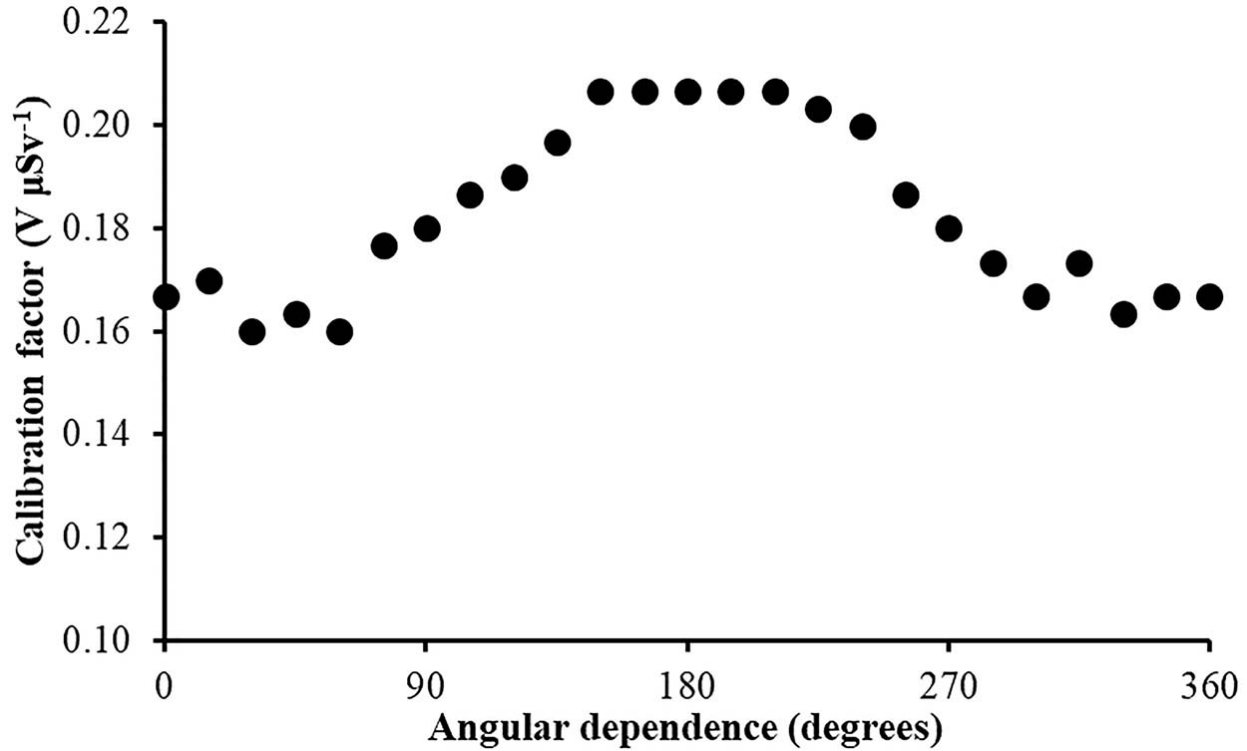
Angular dependence of calibration factors (electret potential drop per μSv) for LST electret ionization chambers irradiated with gamma rays (point source ^137^Cs) (with an ambient dose equivalent value for each irradiation of 300 μSv).

**Figure 10:**
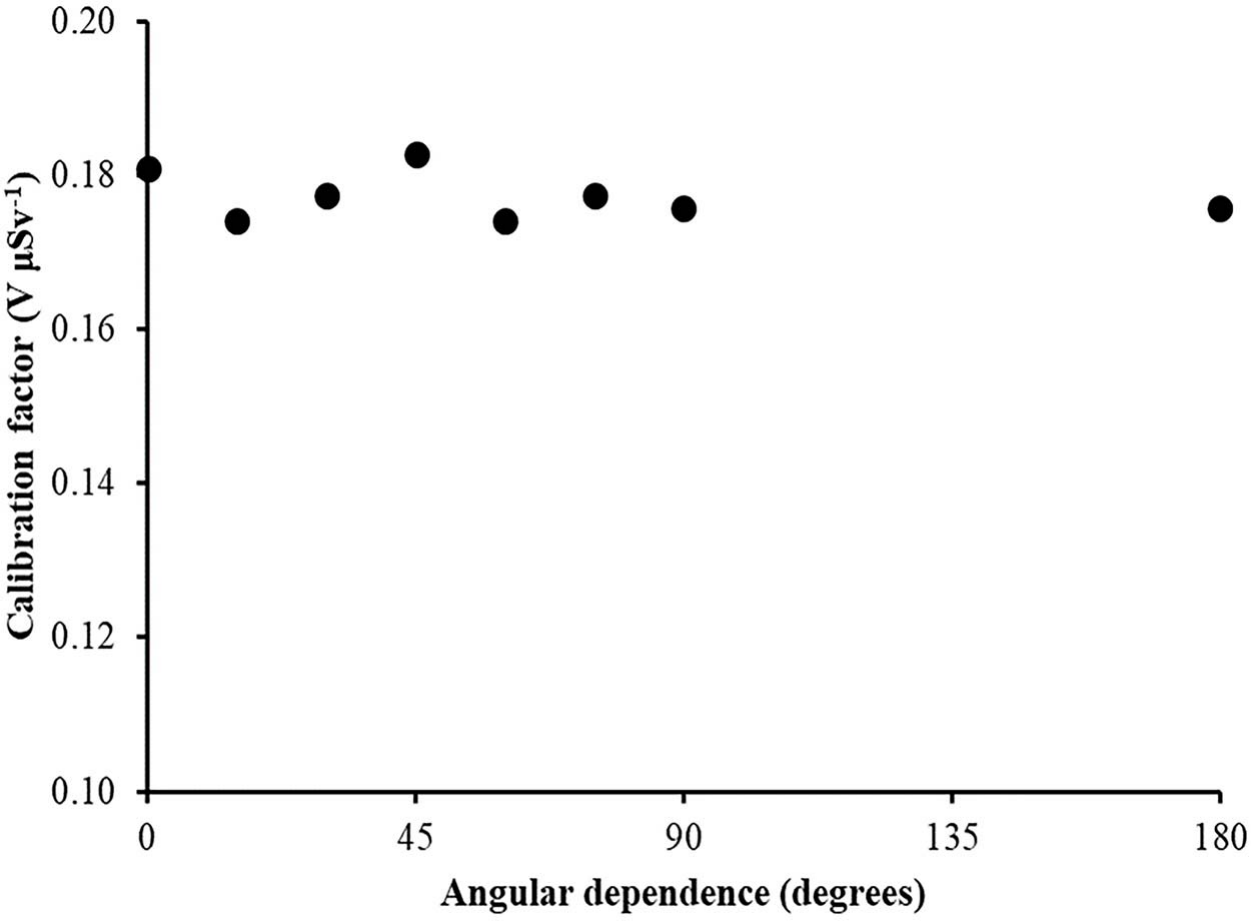
Angular dependence of calibration factors (electret potential lowering per μSv) for LST electret ionization chambers irradiated with gamma rays (point source ^60^Co) (with an ambient dose equivalent value for each irradiation of 290 μSv).

In Figures 7–10, the angular dependence of the calibration factors (electret’s voltage drop due to irradiation in terms of ambient dose equivalent) for four different irradiation energies is shown. For most incident photon energies (except gamma rays from ^60^Co source), an angular dependence of the calibration factors is observed.

### Results of the field measurements

In total, 110 EICs were installed in the eight locations shown in Figure 1, for three consecutive time periods: 38 EIC during November 2017–April 2018, 40 EIC during April 2018–September 2018 and 32 EIC during September 2018–March 2019. About 25 EIC (22.7% of the total number) performed an abnormal discharge and were disregarded in the evaluation. In the same locations, ambient dose equivalent rate was measured with portable HPGe detectors and Reuter-Stokes HPIC. The number of *in situ* measurements performed with HPGe detectors in each location was shown in Table 1. For the HPIC, dose rate is calculated on 10-minute intervals, and data are stored in 1-hour intervals. The mean ${\dot{H}}^{\ast }$ values measured with HPGe and HPIC, respectively, in the eight locations are compared in Table 3. The uncertainties presented in Table 3 are expanded uncertainties with a coverage factor *K* = 1. It is clearly observed that the mean ${\dot{H}}^{\ast }$ values measured with HPIC detectors for the three time periods coincide within the uncertainties of each other in every location.

**Figure 11:**
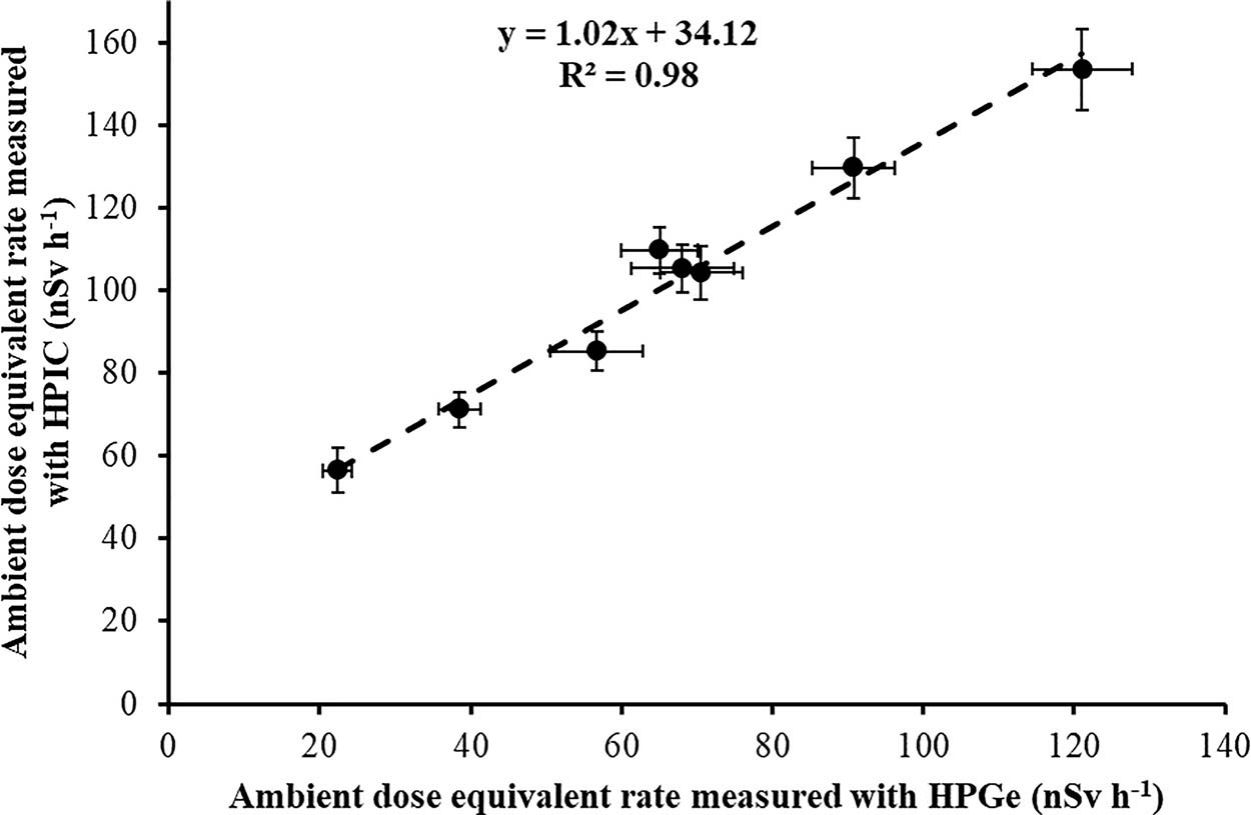
Correlation between mean ambient dose equivalent rate measurements in the eight locations performed by the two detectors (HPIC and HPGe).

In Figure 11, the correlation between the mean values of the dose rate measurements in the eight locations performed by the two detectors (HPIC and HPGe) during the years 2017–2019 is shown. A strong (*R*^2^ = 0.98) linear correlation with a slope of about 1.02 and a constant value of 34 nSv h^−1^ is found between the mean values of the dose rate measurements performed by the two detectors (HPIC and HPGe). The slope of the linear correlation indicates a difference of only 2% between the terrestrial gamma dose rates measured by the two instruments (HPIC and HPGe). The constant value of 34 nSv h^−1^ is very similar to the ambient dose equivalent rate due to cosmic radiation (excluding neutrons) at sea level (HPIC detectors are sensitive to gamma and comic radiation; on the contrary, HPGe detectors are sensitive only to gamma radiation). Wissmann studied^(19)^ the time variation of the ambient dose equivalent rate due to cosmic radiation. He found an ${\dot{H}}^{\ast }$ value of 33 nSv h^−1^ due to cosmic radiation (excluding neutrons) at ground level with an absolute variation of ±2.6 nSv h^−1^ which is very similar to our 34 nSv h^−1^ measured value. Normally, HPIC detectors have an over response to cosmic radiation, and therefore, one could consider this very good agreement as a coincidence. However, in a very recent work^(14)^, the Reuter-Stokes ionization chambers of the Greek Early Warning System Network have been recalibrated to measure in terms of ambient dose equivalent rate and in such a way that two major concerns were corrected^(14)^: (1) any calibration issue of the Reuter-Stokes HPIC detectors due to their home-made calibration in terms of exposure and (2) the well-known over response^(15)^ of the Reuter-Stokes HPIC detectors to cosmic radiation.

**Figure 12:**
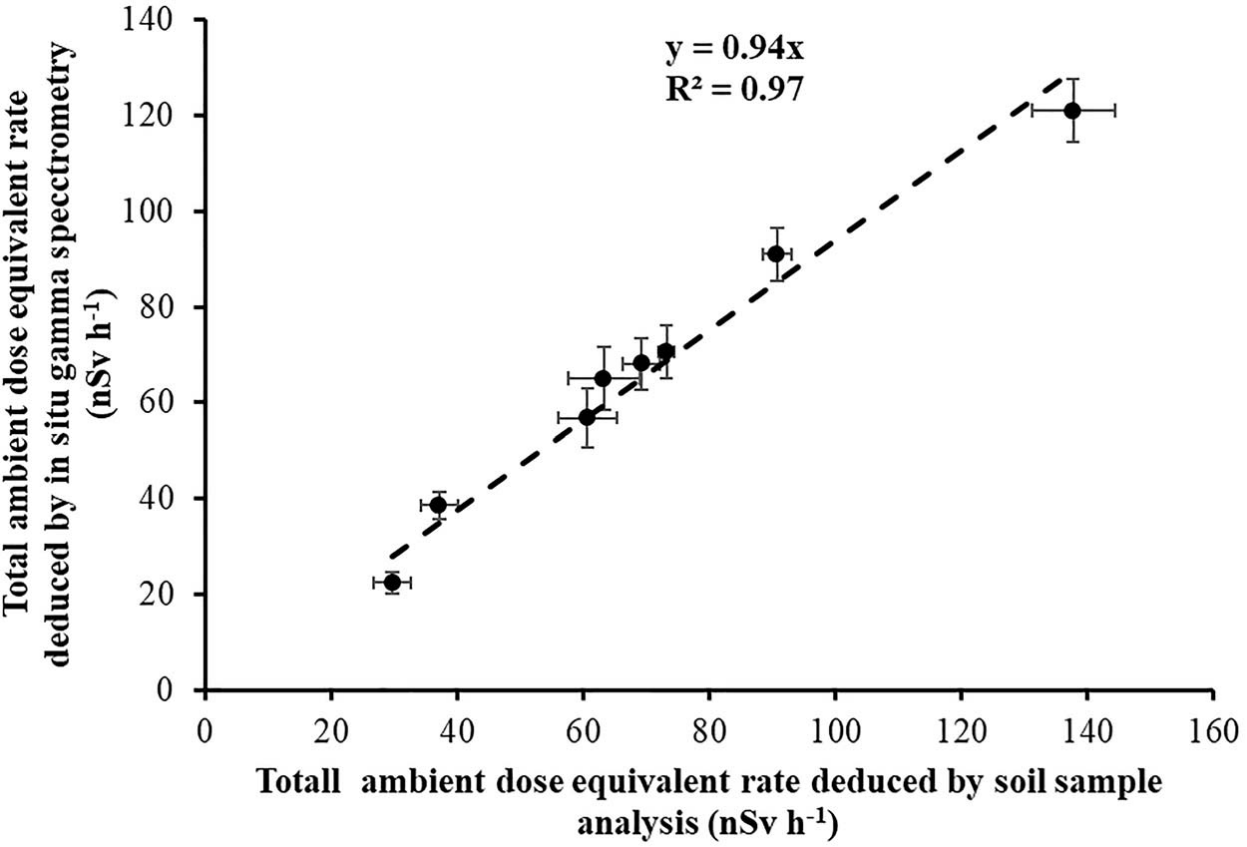
Correlation between the mean values of the total gamma dose rate measurements in the eight locations deduced by in situ gamma spectrometry measurements and indirectly by soil sample analysis.

The total gamma ambient dose equivalent rate is the sum of the gamma dose rates due to (1) uranium series, (2) thorium series, (3) ^40^K and (4) ^137^Cs (due to the Chernobyl accident). In Table 4, the gamma dose rates of the different components of the total dose rate as measured *in situ* and as deduced by soil sample analysis are compared. A good agreement between direct (*in situ* gamma spectrometry) and indirect (from soil sample analysis) measurements of the dose rates is observed despite the fact that *in situ* gamma spectrometry measurement covers a soil area of 10-m radius and the soil samples corresponded to soil layers of an area of only 38 cm^2^ and a depth of 5 cm. In both methods (direct and indirect), what is measured is the activity of natural gamma emitters and ^137^Cs (from Chernobyl accident) in the soil. From the measured activities, ambient dose equivalent rates 1 meter above soil are deduced using the conversion coefficients calculated by Lemercier *et al.*^(18)^, which corresponds to a soil volume of 1500-m radius and 1.5-m depth. Therefore, the good agreement between the two methods is due to the fact that natural gamma emitters are, in principle, homogeneously distributed around the detector. In Figure 12, the correlation between the total dose rates deduced directly by *in situ* gamma spectrometry measurements and indirectly by soil sample analysis is presented. The mean difference between direct (*in situ* gamma spectrometry) and indirect measurements is about 6%.

**Figure 13:**
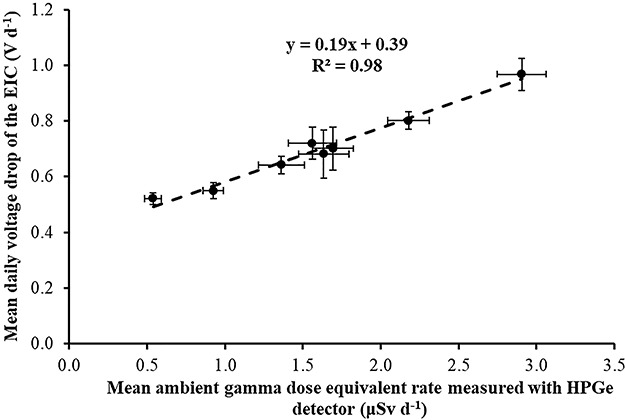
Correlation between mean daily electret potential lowering (in Volts) with the mean daily ambient dose equivalent, measured with portable HPGe detector in the eight locations for the first time period (November 2017–April 2018).

**Figure 14:**
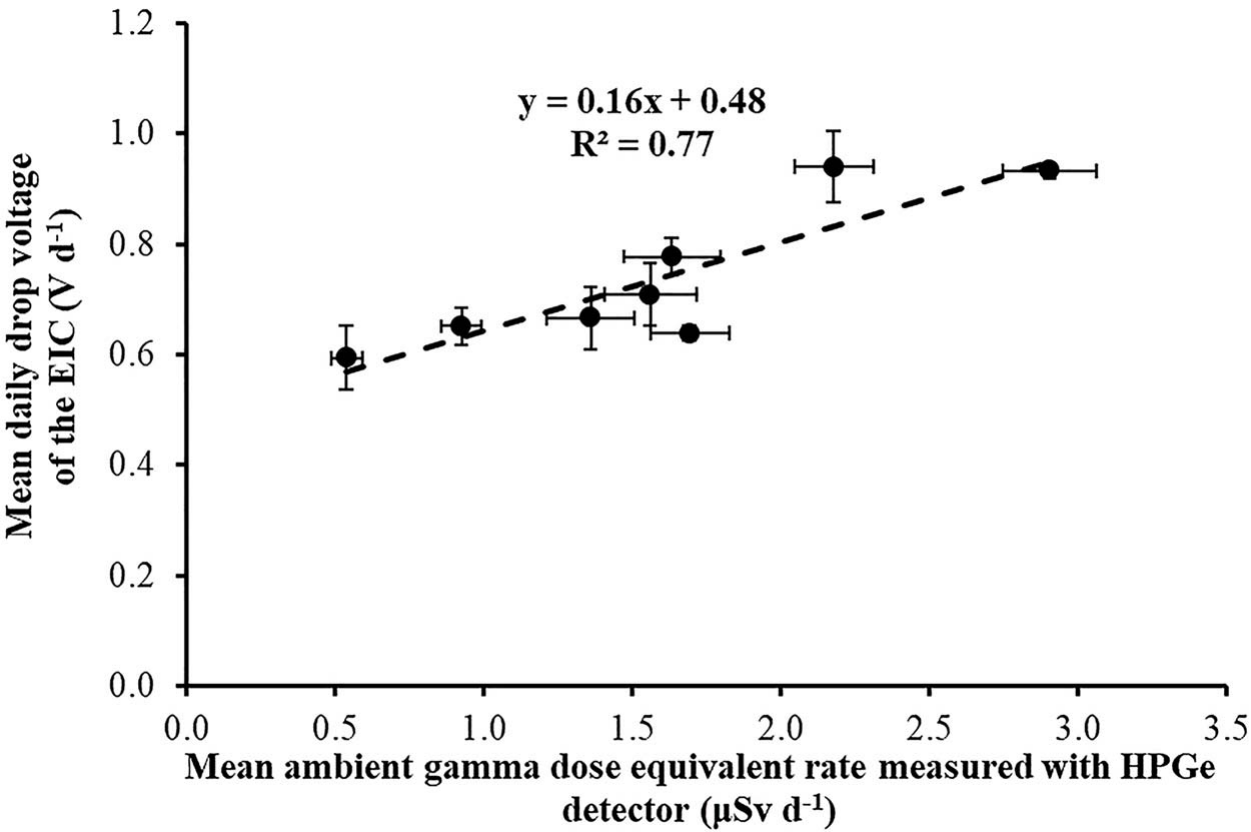
Correlation between mean daily electret potential drop (in Volts) with the mean daily ambient dose equivalent, measured with portable HPGe detector in the eight locations for the second time period (April 2018–September 2018).

**Figure 15:**
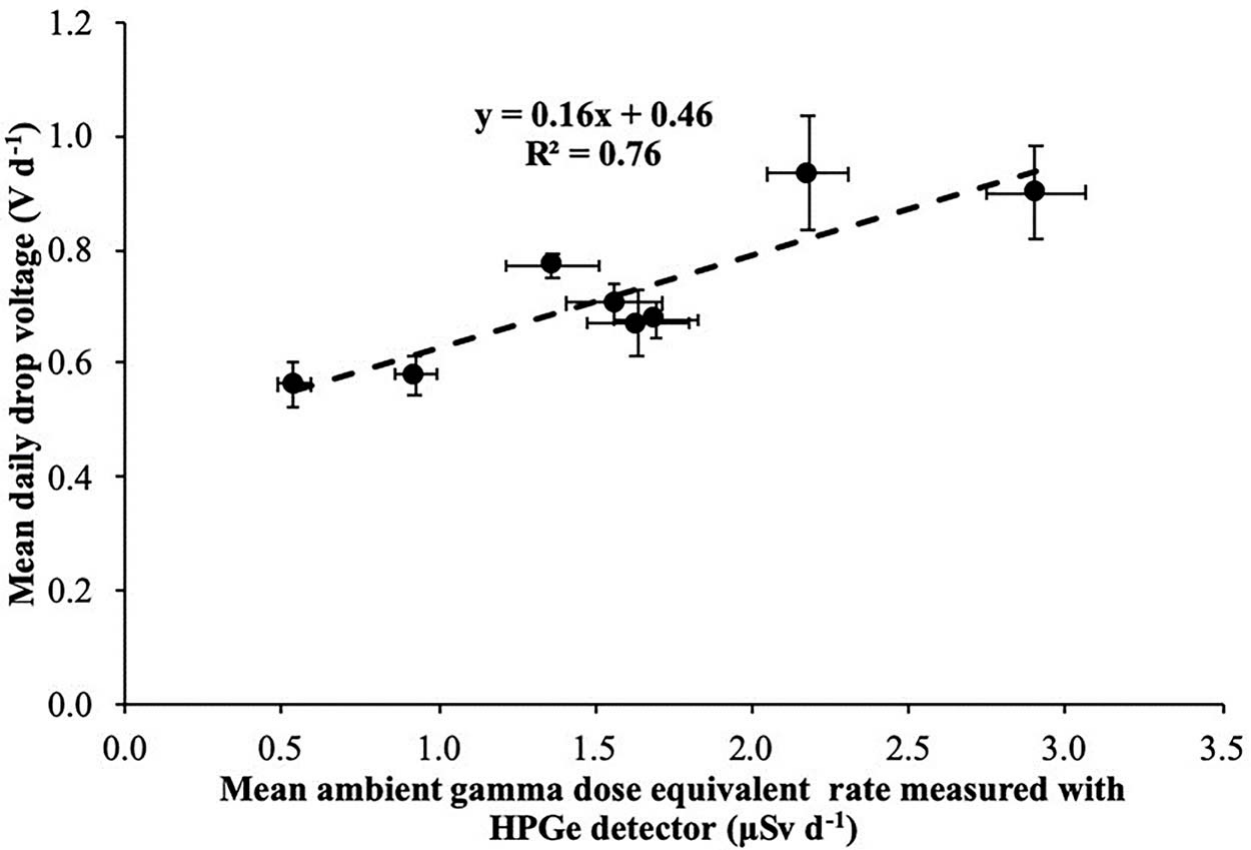
Correlation between mean daily electret potential drop (in Volts) with the mean daily ambient dose equivalent, measured with portable HPGe detector in the 8 locations for the third time period (September 2018–March 2019).

**Figure 16:**
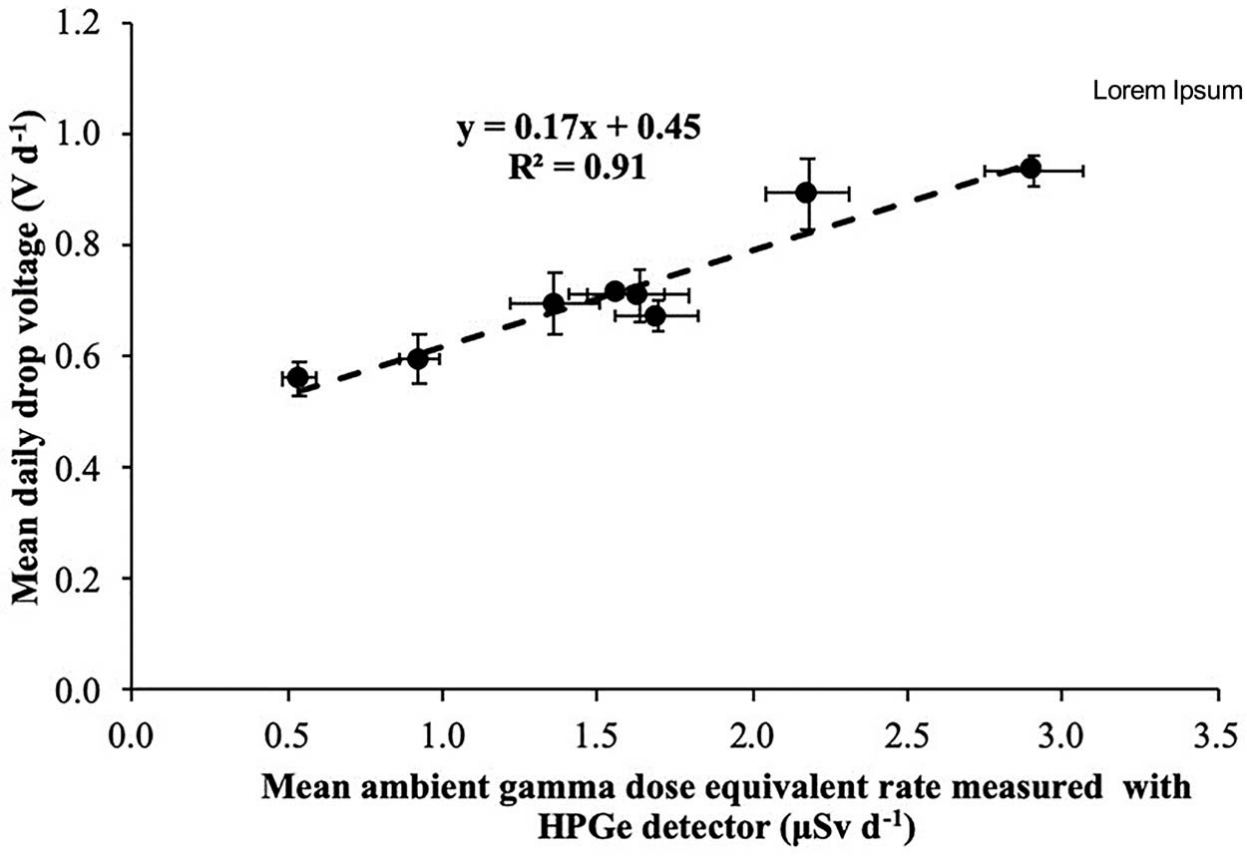
Correlation between mean (for all time periods) daily electret potential lowering (in Volts) with the mean daily ambient dose equivalent, measured with portable HPGe detector in the eight locations.

**Figure 17:**
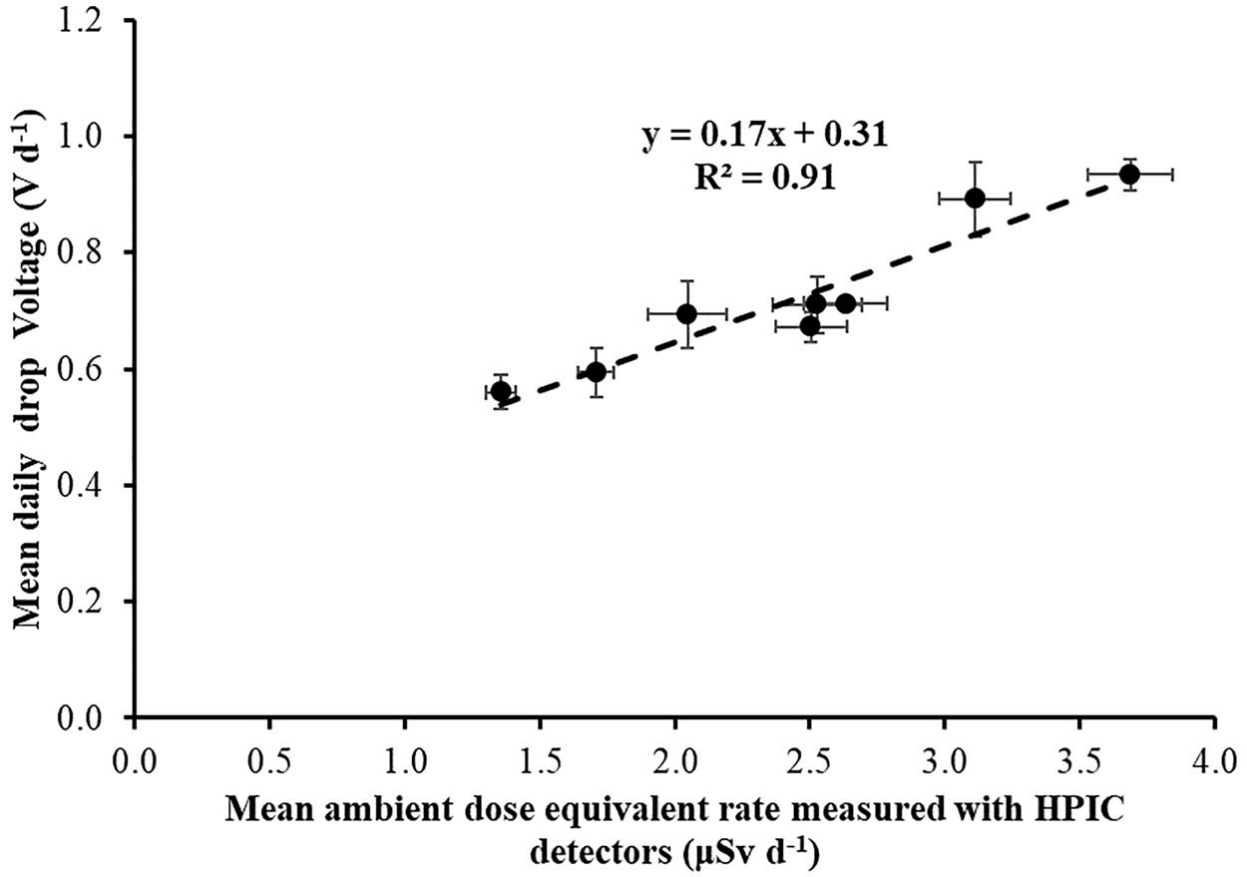
Correlation between mean (for all time periods) daily electret potential lowering (in Volts) with the mean daily ambient dose equivalent, measured with HPIC detectors in the eight locations.

**Figure 18:**
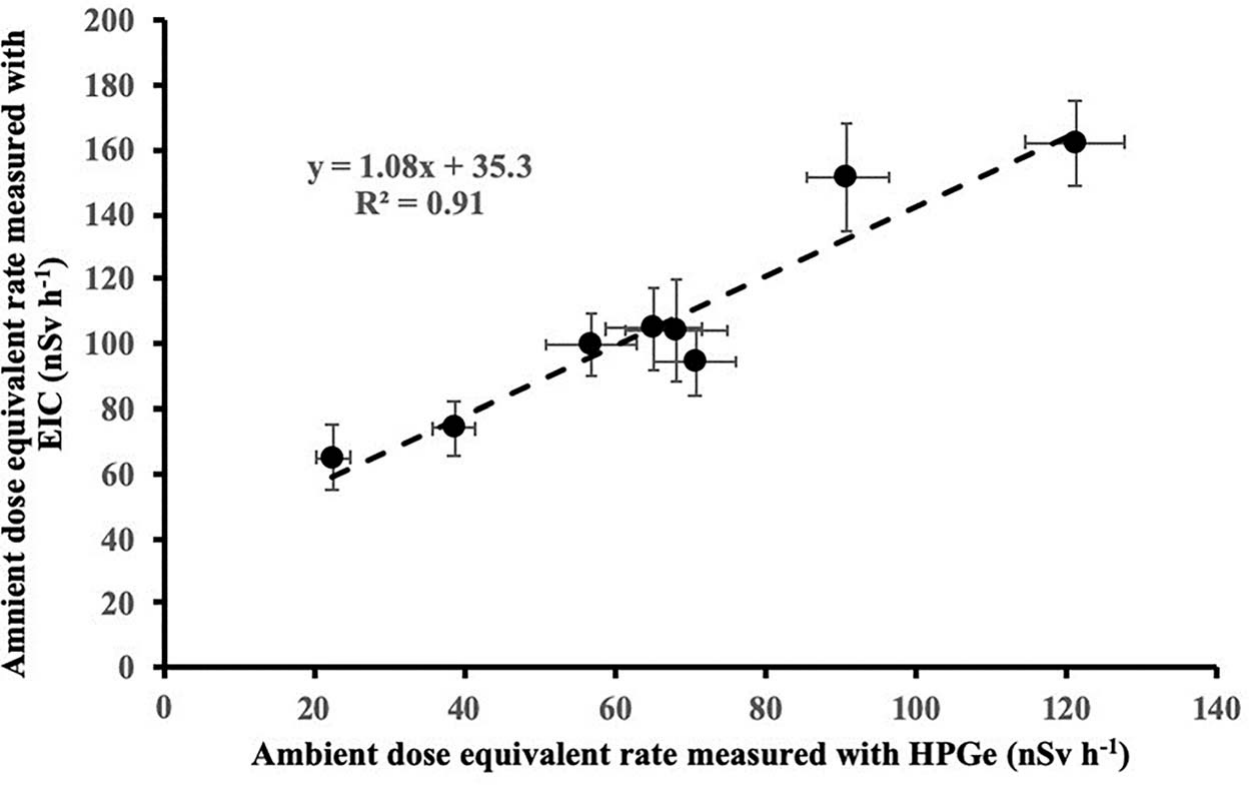
Correlation between mean ambient dose equivalent rate measurements in the eight locations performed by the two type detectors (EIC and HPGe).

**Table 3:** Mean ambient dose equivalent rate (${\dot{H}}^{\ast }$) measured in each location with HPGe detectors and Reuter-Stokes HPIC.

	HPGe nSv h^−1^	HPIC nSv h^-1^		
Location	All periods	1st period November 2017–April 2018	2nd period April 2018–September 2018	3rd period September 2018–March 2019
Thessaloniki	38.6 ± 2.8	67.8 ± 3.5	73.3 ± 4.9	72.5 ± 4.1
Serres	121.0 ± 6.5	146.3 ± 5.0	148.0 ± 13.0	166.5 ± 11.6
Alexandroupoli	56.7 ± 6.1	88.7 ± 5.7	85.8 ± 3.7	81.2 ± 4.8
Komotini	65.0 ± 6.5	108.2 ± 6.5	111.2 ± 4.7	109.7 ± 5.2
Volos	22.3 ± 2.2	54.6 ± 6.0	56.1 ± 5.4	58.8 ± 5.1
Kavala	91.0 ± 5.5	127.2 ± 7.1	128.6 ± 7.3	133.2 ± 7.5
Ptolemaida	70.5 ± 5.5	104.2 ± 5.6	105.1 ± 6.2	103.6 ± 7.6
Ioannina	68.1 ± 5.5	109.3 ± 6.0	104.9 ± 5.5	101.7 ± 5.2

**Table 4:** Dose rate due to the different components of the total gamma dose rate and their contribution, as deduced by the *in situ* gamma spectrometry measurements and from soil sample analysis in the eight locations.

Location	Uranium series (nSv h^−1^)		Thorium series (nSv h^−1^)		^40^K (nSv h^−1^)		^137^Cs (nSv h^−1^)		Total (nSv h^−1^)	
	*In situ*	Indirect	*In situ*	Indirect	*In situ*	Indirect	*In situ*	Indirect	*In situ*	Indirect
Thessaloniki	**7.8 ± 1.6 (20.3%)**	7.3 ± 1.0 (19.6%)	**10.3 ± 1.6 (26.7%)**	8.9 ± 1.3 (23.9%)	**9.0 ± 1.4 (23.2%)**	9.8 ± 1.2 (26.3%)	**11.5 ± 1.8 (29.8%)**	11.2 ± 2.1 (30.1%)	**38.6 ± 2.8**	37.2 ± 2.9
Serres	**30.5 ± 2.5 (25.2%)**	34.3 ± 3.0 (24.9%)	**49.0 ± 2.9 (40.5%)**	49.1 ± 4.3 (35.6%)	**38.3 ± 2.1 (31.7%)**	51.8 ± 24.0 (37.5%)	**3.2 ± 0.3 (2.6%)**	2.7 ± 1.0 (2.0%)	**121.0 ± 6.5**	137.8 ± 6.6
Alexandroupoli	**12.2 ± 1.4 (21.5%)**	14.0 ± 3.7 (23.1%)	**23.1 ± 2.7 (40.7%)**	19.7 ± 1.7 (32.5%)	**20 ± 2.3 (35.3%)**	25.3 ± 2.4 (41.7%)	**1.4 ± 0.2 (2.5%)**	1.7 ± 0.3 (2.8%)	**56.7 ± 6.1**	60.7 ± 4.7
Komotini	**15.4 ± 2.2 (23.7%)**	16.3 ± 2.6 (25.8%)	**23.8 ± 2.3 (36.6%)**	18.6 ± 3.2 (29.4%)	**25.3 ± 2.2 (38.9%)**	27.5 ± 4.0 (43.4%)	**0.5 ± 0.1 (0.8%)**	0.9 ± 0.4 (1.4%)	**65.0 ± 6.5**	63.3 ± 5.7
Volos	**6.7 ± 1.0 (30.0%)**	7.2 ± 2.4 (24.3%)	**7.1 ± 0.9 (31.9%)**	8.4 ± 0.6 (28.2%)	**8.5 ± 0.9 (38.1%)**	14.1 ± 1.4 (47.5%)	**− (0%)**	−(0%)	**22.3 ± 2.2**	29.7 ± 2.9
Kavala	**20.7 ± 1.5 (22.8%)**	18.6 ± 2.2 (20.5%)	**37.4 ± 3.1 (41.1%)**	31.0 ± 0.3 (34.1%)	**32.5 ± 2.8 (35.7%)**	40.3 ± 0.4 (44.4%)	**0.4 ± 0.1 (0.4%)**	0.9 ± 0.2 (1.0%)	**91.0 ± 5.5**	90.8 ± 2.3
Ptolemaida	**14.3 ± 1.8 (20.3%)**	16.2 ± 0.8 (22.1%)	**25.6 ± 1.3 (36.3%)**	20.3 ± 0.5 (27.7%)	**22.5 ± 3.2 (31.9%)**	25.3 ± 0.9 (34.6%)	**8.1 ± 0.6 (11.5%)**	11.4 ± 0.4 (15.6%)	**70.5 ± 5.5**	73.2 ± 1.3
Ioannina	**21.4 ± 4.1 (31.4%)**	20.6 ± 1.0 (29.7%)	**33.2 ± 2.5 (48.8%)**	30.7 ± 2.1 (44.3%)	**13.3 ± 0.7 (19.5%)**	17.7 ± 1.8 (25.5%)	**0.2 ± 0.1 (0.3%)**	0.3 ± 0.1 (0.4%)	**68.1 ± 5.5**	69.3 ± 3.0

The mean daily electret potential drop (in Volts) was compared with the mean daily ambient dose equivalent, measured with portable HPGe detector and Reuter-Stokes HPICs. In Figures 13–15, the mean daily electret potential drop (in Volts) is correlated with the mean daily ambient dose equivalent, measured with the portable HPGe detector in the eight locations for the three time periods November 2017–April 2018 (Figure 13), April 2018–September 2018 (Figure 14) and September 2018–March 2019 (Figure 15).

The slope of the linear equations shown in Figures 13–15 is due to gamma radiation (HPGe detectors are only sensitive to gamma radiation; on the contrary, electret ionization chambers are also sensitive to cosmic radiation). The slopes are the ‘field’ gamma calibration factors (electret’s voltage drop due to gamma radiation in terms of ambient dose equivalent in V μSv^−1^). A ‘field’ calibration factor of 0.19 V μSv^−1^ was deduced from the first period of measurement and 0.16 V μSv^−1^ from the second and third periods of measurement. In Figure 16, the correlation between the mean (for all time periods) daily electret potential lowering (in Volts) with the mean daily ambient dose equivalent, measured with the portable HPGe detector in the eight locations, is shown. A mean ‘field’ calibration factor of 0.17 V μSv^−1^ is deduced from the three time periods, which is in good agreement with the 0.16 V μSv^−1^ deduced from the Laboratory studies in the IRCL of the GAEC.

The constant value of 0.45 V d^−1^, shown in Figure 16, must be related also to cosmic radiation at least partially. As mentioned previously, HPGe detectors are only sensitive to gamma radiation; on the contrary, electret ionization chambers are also sensitive to cosmic radiation. It is therefore interesting to correlate (Figure 17) the mean (of all time periods) daily electret potential drop (in Volts) with the mean daily ambient dose equivalent, measured with Reuter-Stokes HPIC in the eight locations, as both detectors EIC and HPIC are sensitive to gamma and cosmic radiation.

Again, a mean ‘field’ calibration factor of 0.17 V μSv^−1^ is deduced from the three time periods, in perfect agreement with the one deduced by *in situ* gamma spectrometry with the portable HPGe detector and a very good agreement with the 0.16 V μSv^−1^ deduced from the Laboratory studies. In principle, the constant value of 0.31 V d^−1^ should not be expected as both detectors (EIC and HPIC) are ionization chambers and therefore sensitive to cosmic radiation. However, it is, known from previous studies^(10)^, a consistent over-response by the EICs with respect to co-located HPICs. The Idaho National Laboratory operated in the past^(10)^, an environmental gamma radiation detection network consisting of a series of HPICs to provide real-time ambient radiation measurements and a series of passive environmental EICs (similar type as in the present work) to increase coverage area and measure cumulative dose over a calendar quarter. The Idaho Department of Environmental Quality INL Oversight Program identified a consistent over-response of ~40% by the EICs with respect to co-located HPICs. This over-response is likely attributable to several factors, including inherent voltage loss by the electret material not due to ionization within the chamber^(10)^. An electret voltage loss of 0.14–0.34 V d^−1^ was identified^(10)^ with a mean electret voltage loss of 0.2 ± 0.09 V d^−1^. When this voltage correction was applied^(10)^ to environmental data, EIC response was within 10% of the co-located HPIC response. In the present work, a voltage correction of 0.31 V d^−1^ should be applied in order to the EIC’s response to be in accordance with the co-located HPIC response. This voltage correction of 0.31 V d^−1^ found in the present work is within the electret voltage loss range of 0.14–0.34 V d^−1^ identified^(10)^ by Jones and Paulus in 2008.

Applying to the EIC’s data:

(1) the voltage correction of 0.31 V d^−1^ due to inherent voltage loss by the electret material and not due to ionization within the chamber; and
(2) the calibration factor (electret’s voltage drop due to irradiation in terms of ambient dose equivalent) of 0.16 V μSv^−1^ as measured in the IRCL of the GAEC.

The mean ambient dose equivalent rate measured with the EICs in the different locations could be deduced and compared (Figure 18) with those measured by the portable germanium detectors (HPGe). A strong (*R*^2^ = 0.91) linear correlation with a slope of about 1.08 and a constant value of 35 nSv h^−1^ is found between the mean values of the dose rate measurements performed by the two type of detectors (EIC and HPGe). The slope of the linear correlation indicates a difference of less than 10% between the terrestrial gamma dose rates measured by the two instruments (EIC and HPGe). The constant value of 35 nSv h^−1^ is similar to the ambient dose equivalent rate due to cosmic radiation (excluding neutrons) at sea level (33 ± 2.6 nSv h^−1^)^(19)^. It should be noted that EICs are sensitive to gamma and cosmic radiation; on the contrary; HPGe detectors are sensitive only to gamma radiation.

## CONCLUSIONS

The capabilities of EICs to measure mean ambient dose equivalent rates were investigated by performing both laboratory and field studies of their properties.

The main conclusions concerning the laboratory studies are as follows:

(1) The study of the calibration factors (electret potential drop per μSv) as a function of the electret voltage indicated that for electret voltages above 450 V, the calibration factors are constant. On the contrary, for initial electret voltage with less than 450 V, the calibration factors depend on the initial electret voltage. It is therefore recommended to use EIC with relatively high initial electret voltage in case of environmental gamma monitoring measurements.
(2) The calibration factors as a function of the irradiation photon energy (at zero-degree incidence) are clustered within 14% around a mean value of 0.16 V μSv^−1^.
(3) For most incident photon energies (except gamma rays from ^60^Co source), an angular dependence of the calibration factors is observed.

The main conclusions concerning the field studies are as follows:

(1) A mean ‘field’ calibration factor of 0.17 V μSv^−1^ is deduced from the three time periods, in a good agreement with the 0.16 V μSv^−1^ deduced from the Laboratory studies.
(2) In case of long-term environmental gamma monitoring with EIC, at least four EIC should be used in each location due to the possibility of abnormal discharge of the electrets (25 electrets in a total number of 110 electrets had an abnormal discharge and were disregarded for evaluation).
(3) An inherent voltage loss of 0.31 V d^−1^ by the electret material (not due to ionization within the chamber) has been found in accordance with previous results 0.14–0.34 V d^−1^.
(4) The difference between the mean ambient dose equivalent rate measured by EIC detectors and portable HPGe detectors is less than 10%. The mean ambient dose equivalent rate due to cosmic radiation deduced with the EIC detectors is 35 nSv h^−1^, which is like the ambient dose equivalent rate due to cosmic radiation (excluding neutrons) at sea level (33 ± 2.6 nSv h^−1^).
(5) The correlation between mean ambient dose equivalent rate measurements in the eight locations performed by portable HPGe and Reuter-Stokes HPIC indicated a difference of only 2% between the terrestrial gamma dose rates measured by the two instruments (HPIC and HPGe). In addition, a constant value of 34 nSv h^−1^ is found between the mean values of the dose rate measurements performed by the two detectors (HPIC and HPGe). The constant value of 34 nSv h^−1^ is very similar to the ambient dose equivalent rate due to cosmic radiation (excluding neutrons) at sea level.
(6) Last but not least, a good agreement between direct (*in situ* gamma spectrometry) and indirect (from soil sample analysis) measurements of the ambient dose equivalent rates was observed. The mean difference between direct (*in situ* gamma spectrometry) and indirect measurements is about 6%.

## FUNDING

This work was performed in the framework of the project 16ENV04 ‘Preparedness’ that has received funding from the EMPIR programme co-financed by the Participating States and from the European Union’s Horizon 2020 research and innovation programme.
